# Factors Affecting Continued Use of Subcutaneous Depot Medroxyprogesterone Acetate (DMPA-SC): A Secondary Analysis of a 1-Year Randomized Trial in Malawi

**DOI:** 10.9745/GHSP-D-18-00433

**Published:** 2019-03-22

**Authors:** Holly M. Burke, Mario Chen, Mercy Buluzi, Rachael Fuchs, Silver Wevill, Lalitha Venkatasubramanian, Leila Dal Santo, Bagrey Ngwira

**Affiliations:** a FHI 360, Durham, NC, USA.; b College of Medicine, University of Malawi, Blantyre, Malawi.

## Abstract

**Objective:**

To assess the supply- and demand-side factors influencing continued use of the injectable contraceptive subcutaneous depot medroxyprogesterone acetate (DMPA-SC).

**Methods:**

We conducted a 12-month randomized controlled trial in Malawi to measure DMPA-SC continuation rates. A total of 731 women presenting to clinic-based providers (CBPs) at 6 Ministry of Health clinics or to community health workers (CHWs) in rural communities were randomized to receive DMPA-SC administered by a provider or be trained to self-inject DMPA-SC. Data collectors contacted women after the reinjection window at 3, 6, and 9 months to collect data on discontinuation and women's experiences. Twelve months after enrollment or at early discontinuation, women had their final interview, including pregnancy testing. We compared continuation, pregnancy, and safety by whether DMPA-SC or self-injection training was provided by CHWs versus CBPs. We also conducted an exploratory analysis assessing the association between women's sociodemographic factors and the risk for discontinuation using stratified Cox proportional hazards models.

**Findings:**

The type of provider did not seem to influence continuation, pregnancy, or safety. As reported previously, women in the self-injection group were significantly less likely to discontinue the method compared with women in the provider-administered group (hazard ratio, 0.43; *P*<.001). The risk for discontinuation was also different among health facility catchment sites (*P*<.001). No other assessed sociodemographic factors were found to significantly influence the risk for discontinuation.

**Conclusions:**

Public-sector CHWs can safely and effectively provide DMPA-SC and train women to self-inject DMPA-SC in low-resource settings. DMPA-SC continuation did not seem to be influenced by the type of provider, whether CBP or CHW, or women's sociodemographic characteristics.

## Abstract (precis)

Community health workers can adequately provide DMPA-SC directly or train women on self-injection.

## BACKGROUND

Injectable contraceptives are increasingly popular in low-and middle-income countries and are the predominant modern method used by women in sub-Saharan Africa.^1^ In Malawi, the use of modern contraceptive methods by married women has increased from 7% in 1992 to 58% in 2015–2016; however, unmet need for family planning is considerable at 19%.^2^ Injectables are the most commonly used method—of married women in Malawi who use a modern method of contraception, 30% use injectables. Despite their high use, discontinuation rates for injectables are high; 41% of women of reproductive age have reported discontinuing the method in the first year.^2^ In addition to method-related concerns, travel distance to a nearby health center—over 80% of the approximately 17 million people in Malawi live in rural areas^3^—and frequent contraceptive stock-outs are common barriers to use and continuation.^4^

The World Health Organization (WHO) has endorsed task sharing as a strategy to bridge the human resource gap in the provision of reproductive health services in low-income countries, noting that “task sharing is envisioned to create a more rational distribution of tasks and responsibilities among cadres of health workers to improve access and cost-effectiveness.”^5^ When clinic-based providers (CBPs) share tasks with CHWs, the workload of CBPs is reduced, which allows more time for them to provide higher-level care and curative services while increasing access to contraception for women living in hard-to-reach places—thereby helping to address their unmet family planning needs.^6^

Malawi's program for community-based access to injectable contraception started with a pilot in 2008.^7^ CHWs in Malawi (also called health surveillance assistants) provide health services to catchment areas of approximately 1,000 people each; they are the lowest level of paid government workers.^8^ They have completed secondary school and received 12 weeks of training—the first 8 weeks are in a classroom, followed by 4 weeks of practical training. As is the case in many other low- and middle-income countries, CHWs in Malawi administer intramuscular depot medroxyprogesterone acetate (DMPA-IM) to clients in community settings as part of the family planning method mix they offer.

A subcutaneous (SC) version of DMPA is delivered in a prefilled, auto-disabled Uniject injection system (Sayana Press with 104 mg of medroxyprogesterone acetate in 0.65 mL suspension for injection). DMPA-SC is steadily gaining popularity among family planning users and providers in sub-Saharan Africa as an easy-to-use and accessible contraceptive option. Studies in Senegal and Uganda found that family planning providers preferred the subcutaneous version over the intramuscular formation—providers indicated that DMPA-SC was easier and faster to administer, would decrease stock-outs (due to its all-in-one presentation compared with DMPA-IM, which requires a vial and syringe that may become separated), and would be less painful and therefore preferable for women.^9^ Research has also demonstrated that CHWs can safely provide DMPA-SC in community settings.^10^^,^^11^ Moreover, given the simplified delivery system and subcutaneous administration route, a growing body of evidence underscores the feasibility, acceptability, and efficacy of self-injection of DMPA-SC. Self-injection was found to be acceptable and feasible in Senegal and Uganda.^12^^,^^13^ In the current study, a randomized trial recently conducted in Malawi, self-injection improved 12-month continuation rates significantly compared with provider-administered DMPA-SC, by more than 50% (the primary results are reported elsewhere).^11^ In the Malawi study, both CHWs and CBPs were trained to administer injections and to teach women to self-inject.

A growing body of evidence shows that self-injection of DMPA-SC is feasible and acceptable and that CHWs can safely provide it.

The recent trial in Malawi demonstrated that public-sector family planning providers, including CHWs, can safely provide DMPA-SC and train women to self-inject. However, little is known about whether and how outcomes—including continuation, adverse events, side effects, and pregnancy—vary by supply-side factors such as the type of family planning provider (CHW or CBP) who provides DMPA-SC or self-injection training to women. CBPs in the trial were government nurses and midwives with more health care training than the CHWs, which may influence the quality of services provided. Furthermore, the knowledge base is nascent regarding demand-side factors such as the sociodemographic characteristics of women that may influence 12-month DMPA-SC continuation, especially for self-administered DMPA-SC. The numerous studies that have been conducted to assess determinants of continuation of DMPA-IM indicate that women who receive complete and accurate information on possible side effects are more likely to continue using DMPA^14^^–^^18^ and that side effects, especially menstrual disturbances, are an important factor influencing discontinuation.^16^^,^^19^^–^^23^ In contrast, age, marital status, educational level, and parity have not been shown to significantly impact DMPA-IM continuation.^1^^,^^17^^,^^24^^,^^25^

It is unclear how factors influencing continuation of IM and SC formulations will differ, especially when DMPA-SC is self-administered. Two nonrandomized cohort studies in Burkina Faso and Uganda found no difference in continuation rates between DMAP-SC and DMPA-IM when both were administered by CBPs, but findings showed that increased age and partners' acceptance of family planning increased DMPA continuation in Burkina Faso (no other variables tested with the Uganda data were statistically different).^26^ In acceptability trials conducted with DMPA-IM clients in Senegal and Uganda, most clients preferred DMPA-SC after trying it; the most common reason for this preference was that clients perceived fewer side effects from DMPA-SC compared with DMPA-IM,^10^ though previous safety and effectiveness trials have not demonstrated this difference.^27^^,^^28^ A recent nonrandomized cohort study in Senegal also observed fewer side effects among clients who self-injected DMPA-SC compared with those who received DMPA-IM from a CBP.^29^

Given the limited research on factors affecting continued use of DMPA-SC, especially for self-injected DMPA-SC, the aim of this article is to assess the influence of selected supply- and demand-side factors on continued use of DMPA-SC among Malawian women enrolled in a year-long randomized controlled trial. These data can be used to inform task-sharing decisions and optimize service delivery in Malawi and other low-resource settings.

## METHODS

We used data collected as part of a randomized controlled trial we conducted to compare continuation rates between women who self-inject DMPA-SC and women who receive the same product from a provider. The trial was conducted from September 2015 to February 2017 in 6 Ministry of Health clinics and surrounding communities in rural Mangochi District, Malawi. During the trial, CBPs and CHWs randomized 731 women seeking family planning services to either receive DMPA-SC administered by the provider or be trained to self-inject DMPA-SC. Eligible participants were ages 18 to 40 years, in self-reported good general health, able to understand and willing to sign an informed consent document, willing to give contact information for follow-up, willing to have follow-up visits or interviews, willing to be randomized to the self-injection arm or provider-administered injection group, not pregnant according to WHO guidelines, and able to meet eligibility criteria for receiving DMPA per WHO medical eligibility criteria.^30^^–^^32^ Women in the self-injection group who successfully self-injected at enrollment (assessed by the provider) received 3 doses of DMPA-SC to take home for subsequent self-injections, whereas women in the provider-administered injection group were asked to return to the provider for injections at 3, 6, and 9 months post-enrollment. Data collectors (not providers) contacted women after the reinjection window at 3, 6, and 9 months to collect data on discontinuation and women's experiences. Twelve months after enrollment or at early discontinuation, women had their final interview, including pregnancy testing. Neither participants nor study staff were blinded after randomization; however, the statistical team remained blinded until key decisions for the primary analysis were made. A detailed description of the methods of the randomized controlled trial has been published elsewhere.^11^

Our primary outcome was DMPA-SC discontinuation. Women were considered discontinuers if they did not report receiving an injection within the allowable window of time (12 to 14 weeks after the last injection, according to Sayana Press guidelines). Given that reinjection provides 3 months of protection, participants without a DMPA-SC injection within the window or who were lost to follow-up were considered to have discontinued 3 months after the previous injection. Those who had not discontinued by 12 months were censored at 12 months, when the study ended.

We collected data on adverse events, side effects, and pregnancies occurring throughout the 12-month follow-up. In this article, we report outcomes comparing women assisted by CBPs or CHWs. The analysis of safety data included only participants who successfully received or administered a DMPA-SC injection after randomization.

We estimated Kaplan-Meier cumulative probabilities of contraceptive continuation coverage (with 95% confidence interval [CI] at 3, 6, and 9 months) by provider type and compared the distribution of continuation between these groups using a log-rank test stratified by site using a .05 significance level for a 2-sided comparison. We present these results separately by the original randomization group (i.e., self-injection and provider-administered DMPA-SC) since the primary results demonstrated a large treatment effect on continuation.^11^ We also provide discontinuation incidence estimates and incidence rate ratio with 95% CI comparing the 2 provider types.

We also assessed factors that could potentially influence DMPA-SC discontinuation using Cox proportional hazards models with each of the following covariates: treatment group (i.e., self-injection or provider-administered DMPA-SC), site (i.e., the health facility catchment area where the participant was enrolled), woman's age, marital status, whether she works outside the home, parity, education, religion, previous experience with contraceptives and injectable contraceptives, and whether a CBP or CHW provided DMPA-SC or self-injection training at enrollment.

We assessed each covariate separately and planned to include all covariates found significant at the .05 level in the univariate models in a multivariable model. Except for when we analyzed the effect of site specifically, site was used as a stratification variable in the models as consistent with the randomization scheme. Hazard ratios for discontinuation and 95% CI were provided for each covariate modeled.

The study protocol was reviewed and approved by the Protection of Human Subjects Committee at FHI 360, Durham, NC, USA, as well as the College of Medicine Research and Ethics Committee, University of Malawi. All study staff completed training on research ethics, the protocol, and informed consent administration. All trial participants provided their informed consent to participate. The trial was registered with ClinicalTrials.gov (NCT02293694).

## RESULTS

Participants' sociodemographic characteristics are shown in Table 1. Over 70% were enrolled in the study by a CHW. The mean age was 27 years, and 75% had no schooling or did not complete primary school. Over half were Muslim. Almost all were married or had a sexual partner, and 20% said that their husband or partner did not know about their appointment to receive family planning. Almost all had previously given birth and had 3 living children, on average. The large majority (93%) had previously used contraception, primarily injectables. One-quarter did not want additional children.

**TABLE 1.** Baseline Sociodemographic Characteristics of Participants, September 2015 to February 2017, Mangochi District, Malawi (N=731)

Characteristic	Value
**Provider type at enrollment, No. (%)**	
Clinic-based	205 (28.0)
Community health worker	526 (72.0)
**Age group, years, No. (%)**	
18–24	264 (36.1)
25–29	238 (32.6)
30–35	184 (25.2)
>35	45 (6.2)
**Age, years, mean (SD)**	26.9 (5.2)
**Education, No. (%)**	
No school/less than primary school	545 (74.6)
Completed primary school or higher	185 (25.3)
No response	1 (0.1)
**Religion, No. (%)**	
Christian	310 (42.4)
Muslim	418 (57.2)
None	1 (0.1)
No response	2 (0.3)
**Married or has regular sexual partner, No. (%)**	
Not married and no regular sexual partner	25 (3.4)
Married or regular sexual partner	705 (96.4)
No response	1 (0.1)
**Husband/partner knows respondent receiving family planning today, among those with partner,**^a^ **No. (%)**	
No	137 (20.1)
Yes	522 (76.8)
Don't know	13 (1.9)
No response	8 (1.2)
**Ever given birth, No. (%)**	
No	5 (0.7)
Yes	725 (99.2)
No response	1 (0.1)
**Number of living children, among those who gave birth, No. (%)**	
Less than 3 living children	321 (44.3)
3 or more living children	404 (55.7)
**Number of living children, among those who gave birth, mean (SD)**	3.0 (1.64)
**Would like to have a/another child, No. (%)**	
No	182 (24.9)
Yes	529 (72.4)
Don't know	17 (2.3)
No response	3 (0.4)
**Ever used contraception, No. (%)**	
No	47 (6.4)
Yes	679 (92.9)
No response	5 (0.7)
**Ever used injectables, among those who ever used contraception, No. (%)**	
No	21 (3.1)
Yes	657 (96.8)
No response	1 (0.1)

Abbreviations: No., number; SD, standard deviation.

a This question purposefully excludes 25 women who were married but were not living with their husband and had no other regular sexual partner.

Cumulative probabilities of continuation and 95% CI for each quarter by type of provider at enrollment and treatment group are presented in Table 2. Among women in the self-injection group, the continuation rate through 12 months of contraceptive use was not significantly different for women who received DMPA-SC self-injection training from a CBP [0.79 (95% CI, 0.70 to 0.86)] than those who received the training from a CHW [0.70 (95% CI, 0.64 to 0.75)] (*P*=.77). Though the continuation rates were much lower in the provider-administered group (the self-administered and provider-administered groups had 99 and 199 discontinuations, respectively), we did not find a significant difference between women who received DMPA-SC from a CBP [0.48 (95% CI, 0.39 to 0.57) and those who received the method from a CHW [0.44 (95% CI, 0.38 to 0.50)] (*P*=.78). The incidence rate of discontinuation for those who received self-injection training from a CHW was 9 per 100 injection cycles (95% CI, 7 to 11) compared with 6 per 100 injection cycles (95% CI, 4 to 9) among those who were trained by a CBP. For the provider-administered group, the incidence rate for those who received DMPA-SC from a CHW was 21 per 100 injection cycles (95% CI, 18 to 25) compared with 19 per 100 injection cycles (95% CI, 14 to 25) among those who received the method from a CBP.

There was no significant difference in continuation rates between women who received DMPA-SC self-injection training from clinic-based providers versus CHWs.

**TABLE 2.** Cumulative Probability of Continuation Among Self-Administered and Provider-Administered Clients, Stratified by Type of Provider at Enrollment

Month	Clinic-Based Provider		Community Health Worker	
	Number at Risk	Probability (95% CI)	Number at Risk	Probability (95% CI)
**Self-administered**				
First quarter	97	0.99	267	1.00
Second quarter	96	0.88 (0.79, 0.93)	267	0.86 (0.81, 0.90)
Third quarter	83	0.81 (0.72, 0.88)	226	0.77 (0.71, 0.82)
Fourth quarter	76	0.79 (0.70, 0.86)	202	0.70 (0.64, 0.75)
**Provider-administered**				
First quarter	108	1.00	259	1.00
Second quarter	108	0.69 (0.60, 0.77)	258	0.67 (0.61, 0.73)
Third quarter	74	0.58 (0.48, 0.67)	171	0.53 (0.47, 0.59)
Fourth quarter	59	0.48 (0.39, 0.57)	135	0.44 (0.38, 0.50)

Abbreviation: CI, confidence interval.

The distribution of reasons for discontinuation did not differ significantly by provider type for self-injectors (*P*=.49) or for those in the provider-administered group (*P*=.26). The most common reason for discontinuing was due to missing the reinjection window (data are reported elsewhere^11^). Other reasons for discontinuing (in order of decreasing frequency) included loss to follow-up; by the woman's request, mostly related to side effects of DMPA-SC; and less commonly, by the provider's request for medical reasons. The reasons for discontinuation may underestimate the role of side effects during the trial. This is because after women discontinued, they were no longer counted in the estimates of side effect occurrence as the trial moved forward.

Reasons for discontinuation did not differ significantly by provider type, whether CBP or CHW.

Data from pregnancy tests were incomplete due to refusals, loss to follow-up, and data collectors neglecting to administer a pregnancy test as planned (pregnancy status was unknown for 12% in the self-administered group and 21% in the provider-administered group). Among 612 women tested, 8 pregnancies were identified; 1 with a conception date prior to enrollment and 7 during follow-up. Of the 7 pregnancies, 3 occurred in the self-injection group (1 among CBP clients and 2 among CHW clients) and 4 in the provider-administered group (1 among CBP clients and 3 among CHW clients). Differences observed by type of provider within the self-injection group (*P*>.99) and the provider-administered group (*P*>.99) were not statistically significant.

The percentage of continuing women who experienced side effects decreased over time across all groups (Table 3 and Table 4). The differences in percentages of women experiencing side effects among those trained to self-inject by CBPs compared with those trained by CHWs were not statistically significant: 3 months—20% vs. 28% (*P*=.21), 6 months—15% vs. 18% (*P*=.74), and 9 months—12% vs. 14% (*P*=.70). Similarly, there were no statistically significant differences between those who received DMPA-SC from a CBP compared with a CHW: 3 months—34% vs. 31% (*P*=.61), 6 months—23% vs. 22% (*P*>.99), and 9 months—15% vs. 19% (*P*=.69). Among women who reported side effects, the majority across all groups reported little to no effect on daily life.

The percentage of women experiencing side effects were not significantly different by provider type.

**TABLE 3.** Experience With Side Effects in Last 3 Months Among Self-Administered Participants, Stratified by Type of Provider at Enrollment, No. (%)

	3-Month Follow-Up			6-Month Follow-Up			9-Month Follow-Up		
	CBP	CHW	Overall	CBP	CHW	Overall	CBP	CHW	Overall
**Experienced any side effects or problems over last 3 months?**									
No	74 (79.6)	190 (72.5)	264 (74.4)	72 (84.7)	197 (82.4)	269 (83.0)	68 (88.3)	197 (86.0)	265 (86.6)
Yes	19 (20.4)	72 (27.5)	91 (25.6)	13 (15.3)	42 (17.6)	55 (17.0)	9 (11.7)	32 (14.0)	41 (13.4)
**Type of side effects (among women reporting side effects)**									
Irregular bleeding/spotting	5 (26.3)	14 (19.4)	19 (20.9)	1 (7.7)	4 (9.5)	5 (9.1)	2 (22.2)	5 (15.6)	7 (17.1)
Amenorrhea	10 (52.6)	22 (30.6)	32 (35.2)	7 (53.8)	21 (50.0)	28 (50.9)	5 (55.6)	19 (59.4)	24 (58.5)
Heavy bleeding	3 (15.8)	17 (23.6)	20 (22.0)	1 (7.7)	3 (7.1)	4 (7.3)	0 (0.0)	0 (0.0)	0 (0.0)
Weight gain	1 (5.3)	1 (1.4)	2 (2.2)	0 (0.0)	4 (9.5)	4 (7.3)	0 (0.0)	1 (3.1)	1 (2.4)
Weight loss	1 (5.3)	2 (2.8)	3 (3.3)	1 (7.7)	2 (4.8)	3 (5.5)	0 (0.0)	0 (0.0)	0 (0.0)
Backaches	8 (42.1)	19 (26.4)	27 (29.7)	3 (23.1)	17 (40.5)	20 (36.4)	1 (11.1)	15 (46.9)	16 (39.0)
Headaches	8 (42.1)	21 (29.2)	29 (31.9)	3 (23.1)	14 (33.3)	17 (30.9)	3 (33.3)	10 (31.3)	13 (31.7)
Abdominal pain	7 (36.8)	27 (37.5)	34 (37.4)	4 (30.8)	20 (47.6)	24 (43.6)	3 (33.3)	14 (43.8)	17 (41.5)
Nausea/vomiting	6 (31.6)	12 (16.7)	18 (19.8)	2 (15.4)	4 (9.5)	6 (10.9)	2 (22.2)	5 (15.6)	7 (17.1)
Decreased libido	6 (31.6)	9 (12.5)	15 (16.5)	3 (23.1)	6 (14.3)	9 (16.4)	2 (22.2)	3 (9.4)	5 (12.2)
Soreness at injection site	3 (15.8)	12 (16.7)	15 (16.5)	2 (15.4)	7 (16.7)	9 (16.4)	1 (11.1)	4 (12.5)	5 (12.2)
Skin irritation at injection site	4 (21.1)	3 (4.2)	7 (7.7)	1 (7.7)	7 (16.7)	8 (14.5)	2 (22.2)	9 (28.1)	11 (26.8)
Pain at injection site	7 (36.8)	21 (29.2)	28 (30.8)	1 (7.7)	7 (16.7)	8 (14.5)	1 (11.1)	5 (15.6)	6 (14.6)
Other	4 (21.1)	15 (20.8)	19 (20.9)	3 (23.1)	4 (9.5)	7 (12.7)	3 (33.3)	5 (15.6)	8 (19.5)
**How much did these side effects interfere with daily activities?**									
Not at all	11 (57.9)	46 (63.9)	57 (62.6)	9 (69.2)	37 (88.1)	46 (83.6)	8 (88.9)	30 (93.8)	38 (92.7)
Very little	0 (0.0)	5 (6.9)	5 (5.5)	0 (0.0)	2 (4.8)	2 (3.6)	0 (0.0)	0 (0.0)	0 (0.0)
Little	2 (10.5)	7 (9.7)	9 (9.9)	0 (0.0)	1 (2.4)	1 (1.8)	0 (0.0)	2 (6.3)	2 (4.9)
Moderate	1 (5.3)	4 (5.6)	5 (5.5)	1 (7.7)	1 (2.4)	2 (3.6)	0 (0.0)	0 (0.0)	0 (0.0)
Very much	5 (26.3)	9 (12.5)	14 (15.4)	3 (23.1)	1 (2.4)	4 (7.3)	1 (11.1)	0 (0.0)	1 (2.4)
Don't know	0 (0.0)	1 (1.4)	1 (1.1)	0 (0.0)	0 (0.0)	0 (0.0)	0 (0.0)	0 (0.0)	0 (0.0)

Abbreviations: CBP, clinic-based provider; CHW, community health worker; No., number.

**TABLE 4.** Experience With Side Effects in Last 3 Months Among Provider-Administered Participants, Stratified by Type of Provider at Enrollment, No.(%)

	3-Month Follow-Up			6-Month Follow-Up			9-Month Follow-Up		
	CBP	CHW	Overall	CBP	CHW	Overall	CBP	CHW	Overall
**Experienced any side effects or problems over last 3 months?**									
No	63 (65.6)	169 (68.7)	232 (67.8)	55 (77.5)	143 (78.1)	198 (78.0)	50 (84.7)	125 (81.2)	175 (82.2)
Yes	33 (34.4)	77 (31.3)	110 (32.2)	16 (22.5)	40 (21.9)	56 (22.0)	9 (15.3)	29 (18.8)	38 (17.8)
**Type of side effects (among women reporting side effects)**									
Irregular bleeding/spotting	7 (21.2)	20 (26.0)	27 (24.5)	1 (6.3)	4 (10.0)	5 (8.9)	1 (11.1)	6 (20.7)	7 (18.4)
Amenorrhea	12 (36.4)	20 (26.0)	32 (29.1)	8 (50.0)	14 (35.0)	22 (39.3)	4 (44.4)	10 (34.5)	14 (36.8)
Heavy bleeding	11 (33.3)	13 (16.9)	24 (21.8)	2 (12.5)	8 (20.0)	10 (17.9)	1 (11.1)	4 (13.8)	5 (13.2)
Weight gain	1 (3.0)	4 (5.2)	5 (4.5)	2 (12.5)	4 (10.0)	6 (10.7)	5 (55.6)	3 (10.3)	8 (21.1)
Weight loss	1 (3.0)	3 (3.9)	4 (3.6)	0 (0.0)	2 (5.0)	2 (3.6)	0 (0.0)	3 (10.3)	3 (7.9)
Backaches	11 (33.3)	22 (28.6)	33 (30.0)	5 (31.3)	16 (40.0)	21 (37.5)	3 (33.3)	14 (48.3)	17 (44.7)
Headaches	18 (54.5)	30 (39.0)	48 (43.6)	4 (25.0)	15 (37.5)	19 (33.9)	3 (33.3)	11 (37.9)	14 (36.8)
Abdominal pain	17 (51.5)	34 (44.2)	51 (46.4)	7 (43.8)	13 (32.5)	20 (35.7)	2 (22.2)	9 (31.0)	11 (28.9)
Nausea/vomiting	6 (18.2)	9 (11.7)	15 (13.6)	2 (12.5)	7 (17.5)	9 (16.1)	2 (22.2)	4 (13.8)	6 (15.8)
Decreased libido	5 (15.2)	10 (13.0)	15 (13.6)	5 (31.3)	4 (10.0)	9 (16.1)	4 (44.4)	7 (24.1)	11 (28.9)
Soreness at injection site	2 (6.1)	7 (9.1)	9 (8.2)	0 (0.0)	2 (5.0)	2 (3.6)	1 (11.1)	2 (6.9)	3 (7.9)
Skin irritation at injection site	2 (6.1)	6 (7.8)	8 (7.3)	0 (0.0)	4 (10.0)	4 (7.1)	0 (0.0)	1 (3.4)	1 (2.6)
Pain at injection site	6 (18.2)	16 (20.8)	22 (20.0)	3 (18.8)	8 (20.0)	11 (19.6)	0 (0.0)	3 (10.3)	3 (7.9)
Other	6 (18.2)	8 (10.4)	14 (12.7)	6 (37.5)	7 (17.5)	13 (23.2)	2 (22.2)	6 (20.7)	8 (21.1)
**How much did these side effectsinterfere with daily activities?**									
Not at all	19 (57.6)	52 (67.5)	71 (64.5)	11 (68.8)	31 (77.5)	42 (75.0)	8 (88.9)	22 (75.9)	30 (78.9)
Very little	4 (12.1)	9 (11.7)	13 (11.8)	1 (6.3)	4 (10.0)	5 (8.9)	0 (0.0)	2 (6.9)	2 (5.3)
Little	3 (9.1)	5 (6.5)	8 (7.3)	1 (6.3)	1 (2.5)	2 (3.6)	0 (0.0)	1 (3.4)	1 (2.6)
Moderate	3 (9.1)	4 (5.2)	7 (6.4)	0 (0.0)	4 (10.0)	4 (7.1)	0 (0.0)	2 (6.9)	2 (5.3)
Very much	4 (12.1)	7 (9.1)	11 (10.0)	3 (18.8)	0 (0.0)	3 (5.4)	1 (11.1)	2 (6.9)	3 (7.9)
Don't know	0 (0.0)	0 (0.0)	0 (0.0)	0 (0.0)	0 (0.0)	0 (0.0)	0 (0.0)	0 (0.0)	0 (0.0)

Abbreviations: CBP, clinic-based provider; CHW, community health worker; No., number.

Twenty related or possibly related adverse events were reported by 10 women in the self-administration group (data not shown). Nine of these events were reported by 3 women who received self-injection training by CBPs and 11 of these events were reported by 7 women who received training by CHWs. These differences by type of provider were not statistically significant (*P*=.73 for the differences in proportion of women experiencing adverse events). Twenty-eight related or possibly related adverse events were reported by 17 women in the provider-administered group (data not shown). Nine of these events were reported by 7 women who received DMPA-SC from CBPs and 19 of these events were reported by 10 women who received DMPA-SC from CHWs; these differences were not statistically significant (*P*=.28 for the differences in proportion of women experiencing adverse events). Furthermore, there were no significant differences between the groups in the types of adverse events reported. There were 5 serious adverse events reported during the trial by 4 different women. Two events related to DMPA-SC (menorrhagia and anemia requiring hospitalization) were reported by the same woman in the provider-administered group who was enrolled by a CHW and resolved without sequalae. The other serious adverse events, including 1 death (suspected liver cirrhosis), were unrelated to DMPA-SC.

The results of the Cox model are presented in Table 5. Only treatment group and health facility catchment site were statistically significant predictors of continuation; therefore, no additional multivariable analyses were conducted. Consistent with the primary analysis reported elsewhere, we found that women in the self-injection group were significantly less likely to discontinue compared with women in the provider-administered group (hazard ratio, 0.43; *P*<.001). Risk for discontinuation was also different among clinics (*P*<.001).

Self-injectors were significantly less likely to discontinue DMPA-SC than those who received it from providers.

**TABLE 5.** Baseline Factors That May Influence DMPA-SC Discontinuation (N=731)

Factor	Sample Size^a^	*P* Value	Hazard Ratio (95% CI)
**Self-administered vs. provider-administered**	364 vs. 367	<.001	0.43 (0.33, 0.54)
**Age at enrollment**	731	.18	0.98 (0.96, 1.01)
**Health facility catchment site** ^a^	731	<.001	—
Site 1 vs. Site 6	293 vs. 146	—	2.01 (1.39, 2.89)
Site 2 vs. Site 6	67 vs. 146	—	1.78 (1.09, 2.91)
Site 3 vs. Site 6	90 vs. 146	—	1.75 (1.11, 2.74)
Site 4 vs. Site 6	75 vs. 146	—	2.90 (1.88, 4.47)
Site 5 vs. Site 6	60 vs. 146	—	1.18 (0.66, 2.09)
**Married/regular sexual partner vs. none**	705 vs. 25	.48	0.81 (0.45, 1.45)
**Worked outside home for pay in last 12 months vs. not**	96 vs. 634	.18	1.25 (0.90, 1.72)
**Given birth vs. never given birth**	725 vs. 5	.08	0.42 (0.15, 1.12)
**Completed primary school or higher vs. less or no school**	185 vs. 545	.27	0.86 (0.65, 1.13)
**Christian, none, or other vs. Muslim**	418 vs. 311	.10	1.24 (0.96, 1.61)
**Previous use of contraceptives vs. none or no response**	679 vs. 52	.11	0.72 (0.48, 1.08)
**Previous use of injectables vs. none**	657 vs. 68	.29	0.82 (0.56, 1.19)
**Community health worker vs. clinic-based provider**	526 vs. 205	.45	0.90 (0.68, 1.19)

Abbreviations: CI, confidence interval; DMPA-SC, subcutaneous depot medroxyprogesterone acetate; vs., versus.

a Sample size for each factor varied due to missing values.

b Except for site, the univariable models for all other factors were stratified by site.

## DISCUSSION

Contraceptive continuation is important for reducing unintended pregnancies. This is one of the first studies to explore factors that affect continued use of DMPA-SC through 12 months, including self-administered DMPA-SC. A retrospective study of 2015–2016 Demographic and Health Survey data in Malawi found a 12-month discontinuation rate of 41% for injectable contraceptive users.^2^ In this prospective trial, the discontinuation rates of DMPA-SC through 12 months were 52% and 56%, for clients who received the injections from CBPs and CHWs, respectively. The discontinuation rates for self-injecting clients trained by CBPs and CHWs were substantially lower, 21% and 30%, respectively. The differences in the continuation rates by provider type (CBPs and CHWs) were not statistically different for either self-administered or provider-administered DMPA-SC.

We did not find evidence that the type of provider influenced the risk of discontinuing, pregnancy, or safety, which suggests that CHWs—not only CBPs—can provide DMPA-SC or training on self-injection in low-resource settings without hampering continuation. Wait times at health facilities are often long and health facilities are often overcrowded and understaffed. Permitting CHWs to train women on DMPA-SC self-injection in community settings would enable women to circumvent the long lines and alleviate some of the work load at these health facilities. CHWs are based in rural and low-income areas where there is often high unmet family planning need, and they are more likely to remain in their communities once trained.^33^ In 2009, WHO concluded that CHWs can safely and effectively administer injectable contraceptives in non-clinical settings.^34^ CHW provision of injectable contraception was once innovative but is now a standard of practice. Our results add to the body of evidence supporting task sharing and CHWs' potential to increase access to contraception and reduce unmet family planning needs, despite lower levels of training.^6^ Based on the evidence, self-administered and provider-administrated DMPA-SC should be scaled up in community settings using CHWs.

Permitting CHWs to train women on DMPA-SC self-injection could increase access to contraception and alleviate the work load of other providers.

Of the factors explored, treatment group and health facility catchment site were the only factors that significantly influenced the risk of discontinuation, with self-injection leading to a reduced risk of discontinuation compared with provider administration. Consistent with previous studies of DMPA-IM,^1^^,^^17^^,^^24^^,^^25^ we did not find evidence that sociodemographic factors influenced DMPA-SC continuation. Importantly, we found that education levels did not affect women's ability to self-inject. Most women enrolled in the study had very little education and could inject on time and continue using DMPA-SC during the year-long trial.

Education levels did not affect women's ability to self-inject.

Our findings are consistent with findings from nonrandomized prospective cohort studies in Senegal and Uganda, which observed that clients who self-injected DMPA-SC had a lower risk of discontinuing relative to clients who received DMPA-IM from CBPs.^29^^,^^35^ However, our findings differed from these studies in that they observed several other variables—some that we included in our model and some we did not—that influenced DMPA continuation. In Uganda, rural location and being younger increased discontinuation risk, whereas having a primary or greater education (versus no education), more children, and partner support for family planning increased continuation. In Senegal, paying for travel to the clinic and experiencing side effects increased discontinuation risk, whereas having more education, children, and household assets increased continuation. It may be that our site variable encompasses other underlying factors, such as rurality, which are not otherwise included in our model.

Our results are also similar to a study of provider-administered DMPA-SC in urban Nigeria that found no differences in continuation at 3 months according to the place women received DMPA-SC.^36^ In that study, data were collected from a convenience sample of users who obtained DMPA-SC from selected private-sector providers working in hospital, clinics, and retail drug outlets, as well as licensed Community Health Extension Workers. Unlike our study, the Nigeria study found differences in sociodemographic characteristics: women with some college education or more and those with 4 or more children were more likely to obtain another dose at 3 months. They also found that quality of counseling and side effects influenced continuation.

One limitation of our study is that women's reported outcomes may have been influenced by social desirability bias. Another challenge we faced was missing data for the pregnancy outcome. Given this, our pregnancy data should not be used for estimating the DMPA-SC failure rate. The study was also not designed to assess whether women with different characteristics or being assisted by various types of providers had different risks of discontinuation; therefore, the sample size for some of the comparisons may be too small to be conclusive. Furthermore, these are non-randomized comparisons and may be affected by selection biases. Lastly, there are numerous other variables and combinations of variables that we did not explore but which may influence continuation.

Although we did not find any of the sociodemographic factors associated with DMPA-SC discontinuation to help us target future efforts, the differences observed across sites may indicate the presence of other underlying factors that would be interesting to explore in future studies. For example, providers' management of clients who would like to continue using injectables but arrived late for their scheduled reinjections (although still within the grace period) has been documented to vary and to directly affect clients' continued use of contraception.^37^ Understanding the context and other characteristics of the populations served by these sites is important, but further exploration is not possible in our study due to our sample size and data contents. Despite the site differences, the positive effects of self-injection were present in all sites, which speaks of the robustness of this finding across contexts and further supports our recommendations for scaling up DMPA-SC. Implementation challenges will need to be addressed to make this recommendation possible, including resources and planning for training and advanced provision of commodities for self-administration; however, the introduction and scale-up of this new evidence-based approach addresses the severe shortage of family planning providers and the persistent problem of DMPA discontinuation. We urge WHO and the global health community to expand their endorsements of CHW provision of injectables to include CHW provision of DMPA-SC for self-injection.

## Notes

**Peer Reviewed**

**Funding:** This work was made possible by the generous support of the American people through the U.S. Agency for International Development (USAID) and by the Children's Investment Fund Foundation (CIFF). The contents are the responsibility of FHI 360 and do not necessarily reflect the views of USAID, the U.S. Government, or CIFF. Financial assistance was provided through the Advancing Partners & Communities (APC) agreement funded by USAID under Agreement No. AID-OAA-A-12-00047, beginning October 1, 2012. APC is implemented by JSI Research & Training Institute in collaboration with FHI 360.

**First Published Online:** March 20, 2019
